# Efficient MLC quality assurance using a virtual picket fence test in an MR‐Linac

**DOI:** 10.1002/acm2.70232

**Published:** 2025-09-03

**Authors:** Kai Yuan, Matthew Manhin Cheung, Louis Lee

**Affiliations:** ^1^ Medical Physics Division Department of Medical Innovation & Technology CUHK Medical Centre Hong Kong SAR China; ^2^ Department of Clinical Oncology Faculty of Medicine The Chinese University of Hong Kong Hong Kong SAR China

**Keywords:** log file, MR‐linac, Multi‐leaf collimator, picket fence test, quality assurance

## Abstract

**Background:**

The Elekta Unity MR‐Linac system integrates magnetic resonance imaging (MRI) with a linear accelerator (Linac) for adaptive radiation therapy. Traditional quality assurance (QA) methods for multi‐leaf collimators (MLCs) face challenges in this system due to the magnetic field and limited field size of electronic portal imaging devices (EPID).

**Purpose:**

This study aims to develop a ‘virtual picket fence’ test using machine log files to evaluate MLC performance in the Elekta Unity MR‐Linac system, providing a more efficient and comprehensive QA method that overcomes the limitations of traditional approaches.

**Methods:**

A picket fence test plan with 11 segments was delivered on the Elekta Unity system. Maximum absolute error and root mean square (RMS) error for each leaf were calculated by comparing log file data with nominal values. A deliberate 1 mm error was introduced in selected MLCs to test the sensitivity of the virtual test. The results from the log file were further compared with measurements from radiochromic films.

**Results:**

The maximum deviation between log file data and nominal values was within 1 mm for all leaves. The virtual picket fence test successfully identified MLCs with deviations beyond the 0.5 mm warning threshold in the error‐introduced test. Comparisons with film‐based measurements showed good agreement, with deviations between film and log file data also within 1 mm.

**Conclusions:**

The virtual picket fence test provides an efficient and comprehensive method for MLC QA in the Elekta Unity MR‐Linac system. This method can be integrated into weekly QA workflows alongside traditional film‐based methods for thorough quality control.

## 1 INTRODUCTION

The Elekta Unity MR‐Linac system, developed by Elekta AB in Stockholm, Sweden, represents a state‐of‐the‐art radiation therapy (RT) platform. It integrates a 1.5 Tesla magnetic resonance imaging (MRI) scanner with a 7 MV flattening filter‐free (FFF) linear accelerator (Linac) system.^1^ The Monaco Treatment Planning System (TPS) utilizes Monte Carlo algorithms to precisely model magnetic field effects in dose calculations. This advanced technology enables adaptive treatment plans that respond to daily variations in tumor shape and position, facilitating precise dose delivery while providing real‐time tumor visualization. AAPM TG‐142 emphasizes the importance of periodic quality assurance (QA) for diaphragms and Multi‐leaf collimator (MLC) systems to ensure accurate dose delivery during RT treatments.^2^ Given the Elekta Unity system's utilization of step‐and‐shoot beam delivery, maintaining precise leaf position and reproducibility is crucial. The MV imager and AQUA (Automated Quality Assurance) software offer efficient semi‐automated routines for swift evaluation of MLC and jaw performance. To verify leaf positioning within the field area, it is recommended to irradiate a series of rectangular fields with varying central axis offsets.^3^

**FIGURE 1:**
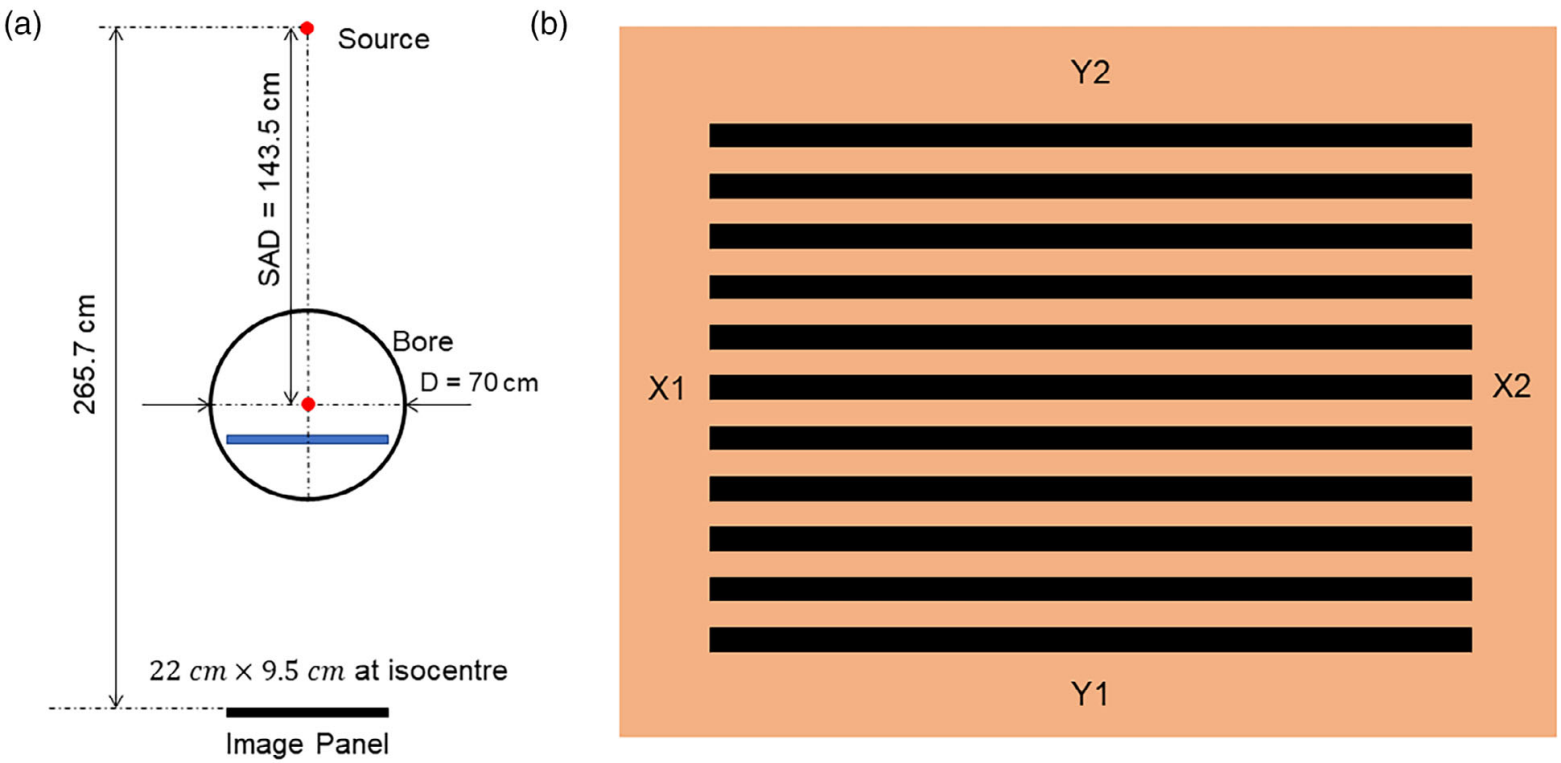
(a) The geometry of the Unity gantry. (b) A demonstration of the picket fence pattern is used. X1 and X2 stand for the X jaw, and Y1 and Y2 stand for MLC. MLC, multi‐leaf collimators.

The Elekta Unity system features a unique geometry compared to conventional radiotherapy devices. It has a source axis distance (SAD) of 143.5 cm and a maximum field size of 57.4 cm × 22.0 cm. The field‐defining diaphragms move across the cross‐plane direction, while 160 MLC leaves move in the in‐plane direction with a nominal pitch of 0.7175 cm.^4^ The imaging panel is positioned 265.7 cm from the source, with its field of view limited to 22.0 cm × 9.5 cm due to cryostat gap constraints (Figure 1a). These geometric characteristics impact QA procedures. The QA routine utilizing electronic portal imaging devices (EPID) and AQUA software can only assess the middle 28 MLCs and is restricted to a limited longitudinal range due to field size limitations.^5^ For a comprehensive monthly or annual MLC performance assessment, expanding the field area is advisable. While a film‐based picket fence test could be adopted to expand the field of view, it presents unique challenges in the Unity system: (1) A single radiochromic film is too small to cover all MLC pairs at the isocenter plane. (2) The strong magnetic field causes picket fence shifts on the irradiated film, complicating absolute MLC position identification. (3) Setting up the film at multiple gantry angles is both challenging and time‐consuming. These factors make film‐based MLC assessment in Unity more demanding than in conventional LINACs.^4^, ^6^, ^7^, ^8^

The Elekta Unity system's log files offer comprehensive data on radiation treatments, including monitor units (MU), gantry angle, and MLC and jaw positions, sampled every 40 ms. These log files serve as valuable resources for conducting QA assessments of MLC performance.^9^, ^10^, ^11^ In this study, we have developed a ‘virtual picket fence test’ utilizing machine log files to evaluate MLC performance. This method involves analyzing the log file generated after delivering a picket fence test plan, eliminating the need for film or EPID. This approach has the potential to serve as a routine monthly check for all MLC pairs, including those that are difficult to assess using the MV imager. The virtual picket fence test offers a novel solution for comprehensive MLC performance evaluation, potentially enhancing the efficiency and scope of QA procedures in the Elekta Unity system.

## 2 METHODS AND MATERIALS

**FIGURE 2:**
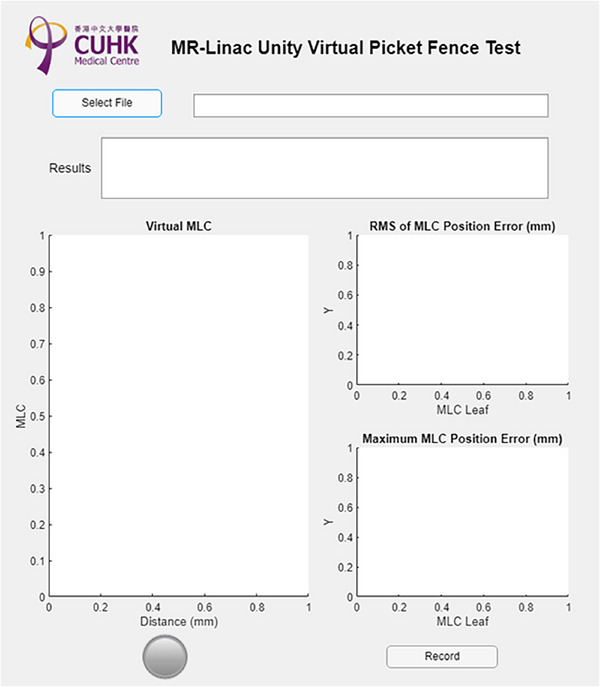
UI for the virtual picket fence test.

In the Elekta Unity beam shaping system, the jaws are positioned only in the transverse direction (X). During beam shaping, two leaf pairs positioned just beyond the jaws function as guard leaves, which remain open slightly outside the treatment field during delivery. In contrast, the longitudinal direction (Y) is shaped exclusively by the MLCs. The jaws, labeled as X1 and X2, operate in the transverse direction, while the MLC banks, designated as Y1 and Y2, adjust the field in the longitudinal direction. For the picket fence test (Figure 1b), a series of 11 different segments are delivered, each with 200 MU. Each segment uses the MLCs to produce a rectangular shape with a nominal size of 1 cm in the *Y*‐axis (longitudinal direction), with a step size of 1 cm. In the *X*‐axis (cross‐plane direction), the jaws remain open, which means the cross‐plane length of each rectangular shape is 57.4 cm. In accordance with TG‐142 guidelines, the tolerance for MLC positioning accuracy is set to a stringent ± 1 mm deviation from the nominal value. The log files can be exported from rich text format (.rtf) to comma‐separated values (.csv) files in a Elekta's proprietary converter, enabling further analysis using in‐house MATLAB (MathWorks, Natick, MA) scripts. To streamline this process, a user‐friendly interface (UI) has been developed for log file analysis, as illustrated in Figure 2.

To evaluate MLC performance, the leaf position readings for each segment were averaged across all sampling points. This allowed us to determine the leaf positions for the $Y_{1,j}^{\log}$​ and $Y_{2,j}^{\log}$​parameters at each of the 11 segments. For each individual leaf, there were 11 different positions corresponding to the 11 segments. The maximum absolute error was then calculated as:

$$\;Y_{i}^{\max}=\;\mathrm{Max}\left(\left|Y_{i,j}^{\log}\;-{\;Y}_{i,j}^{\mathrm{nominal}}\right|\right)$$

where iε{1,2} represents the two leaf banks, jε{1,…,11} represents the 11 different segments, $Y_{i,j}^{\log}$ is the leaf position stored in log file, and $Y_{i,j}^{\mathrm{nominal}}$ is the leaf position set in the picket fence plan.

In addition to calculating the maximum absolute error, we also determined the root mean square (RMS) error of the position error for each leaf across all segments. This provided a comprehensive evaluation of the overall performance of each leaf at different positions. The formula used for the RMS error calculation is:

$$\;Y_{i}^{\mathrm{RMS}}=\sqrt{\frac{1}{11}\sum_{j\;=1}^{11}{\left(Y_{i,j}^{\log}\;-{\;Y}_{i,j}^{\mathrm{nominal}}\right)}^{2}\;}$$

where iε{1,2} represents the two leaf banks, and jε{1,…,11} represents the 11 different segments. An additional picket fence plan was created, deliberately introducing a 1 mm leaf position error in several MLCs to evaluate the sensitivity of the developed QA system in detecting shifted leaf positions. Specifically, a 1 mm leaf position error was introduced in Y1 bank leaves No. 15, 16, and 17; Y2 bank leaves No. 50, 51, and 52; and Y1 leaves No. 63, 64, and 65.

To verify the accuracy of the log file calibration, we irradiated the same correct picket fence test plan on two separate RTQA2 radiochromic films (Ashland, Bridgewater, NJ). The presence of a magnetic field can affect the trajectory of secondary electrons, potentially leading to inaccuracies in determining the actual position of the MLCs on the film. Van Zijp et al. evaluated the effectiveness of using two 2‐mm copper sheets to counteract the prolonged electron trajectory in MR‐Linac.^7^ We also adopted this method to mitigate the electron return effect on the film. Two 2‐mm copper plates were used to cover the film on both the top and back. Two separate films were used, each covering 40 pairs of MLCs on each side.

To quantitatively measure the distance from the leaf end to the isocenter, the isocenter should be defined on the film before irradiation. We used a metal wire to localize the isocenter and employed EPID by irradiating from 0 degree and 90 degree. The isocenter was then marked on the film with a black marker pen. As shown in Figure 5a, the film was placed 14 cm above the couch, corresponding to the level of the isocenter, and solid water pads were used to elevate both the film and copper plate. The irradiated films were scanned using an EPSON 12000XL scanner at a DPI of 200. Only the red channel was used for analysis. For doses below 2 Gy, the red channel demonstrates superior sensitivity for radiochromic films.^12^, ^13^, ^14^ The film was analyzed in MATLAB (MathWorks, Natick, MA). In the X direction, the leaf pairs were localized based on their width and distance from the isocenter marked on the film. In the Y direction, leaf tip positions were determined using the Full Width at Half Maximum (FWHM) of the longitudinal profiles for each leaf. The profiles were smoothed using a moving average filter with the window size of 30 pixels. FWHM analysis for each segment was performed by identifying the maximum and minimum values of each gap. To compare the leaf positions decoded from film with those in the log file, we calculated both the maximum absolute deviation and the RMS. To further validate the results, we analyzed the MLC position accuracy using EPID measurements in the AQUA software. In the test, two segments (4 cm × 20 cm) were shaped by the central 28 pairs of MLCs. The test was delivered in $0^{\circ }$ gantry angle and a non‐zero gantry angle $45^{\circ }$. We calculated the mean, standard deviation (SD), and range for three comparisons: EPID versus nominal leaf positions, log file versus nominal leaf positions, and EPID versus log file measurements.

To ensure the log file analysis was longitudinally stable and robust, we conducted analyses using EPID data and corresponding log files from monthly AQUA tests of MLC positioning. The monthly AQUA tests of MLC positioning are performed at four different angles, spaced 90 degrees apart: 45°, 135°, 225°, and 315°. The same test is conducted weekly at 0°. These data from gantry angles of 0° and 45° over 5 months (T1–T5) were used for longitudinal analysis. Additionally, at time points T1 and T5, we performed picket fence tests using radiochromic film to provide an independent validation of the MLC system's long‐term stability. The testing procedures for the longitudinal analyses are illustrated in Figure 6.

## 3 RESULTS

**FIGURE 3:**
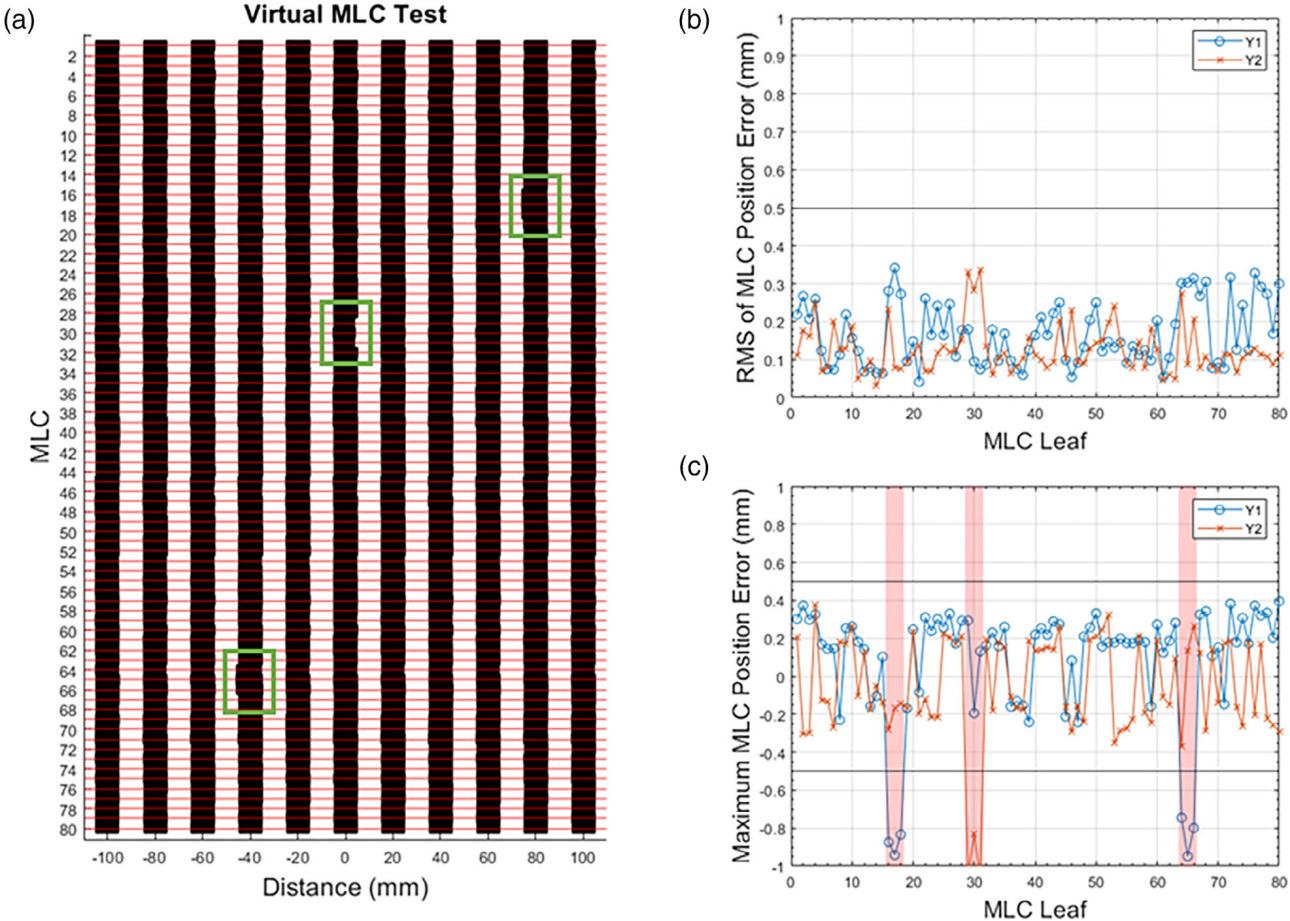
Results from the error introduced picket fence test. (a) Virtual picket fence pattern. (b) RMS of leaf position error for each MLC among 11 segments. (c) Maximum leaf position error for each leaf among 11 segments.

As illustrated in Figure 3a, the absolute positions of the MLCs relative to the isocenter are depicted in the virtual MLC pattern. The position of each MLC pair can be examined within this pattern, where the horizontal red lines represent the longitudinal (Y) trajectory for each pair of MLCs. Each of the 11 black vertical segments simulates a virtual picket fence pattern, akin to an irradiated film. For the picket fence plan with a 1 mm introduced error, MLCs with abnormal leaf positions can be identified. Figure 3b,c demonstrates the RMS and maximum error for each MLC across all 11 segments. Additionally, MLCs exceeding the warning level of 0.5 mm in any segment are highlighted with a red shadow in Figure 3c.

**FIGURE 4:**
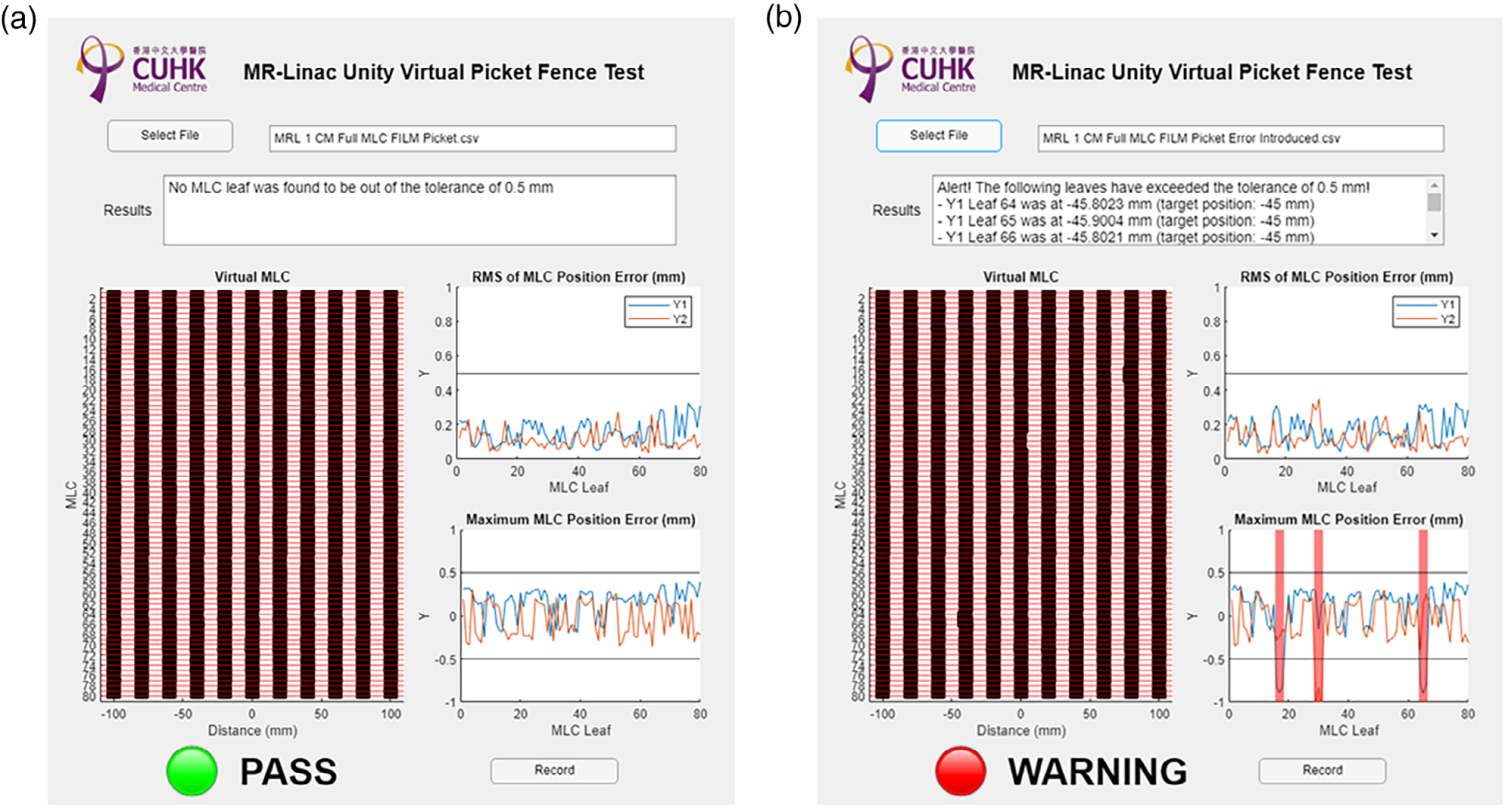
(a) and (b) stand for the PASS and WARNING conditions, respectively.

Figure 4a,b illustrates the absolute positions of the MLC relative to the isocenter, as displayed in the Virtual MLC panel of the UI. In Figure 4a, no MLC exceeded the warning threshold of ± 0.5 mm in the standard picket fence plan. Conversely, Figure 4b demonstrates the successful identification of MLCs with a 1 mm error introduced, encompassing all nine MLCs distributed across three distinct segments. The Virtual MLC panel reports the MLC number, actual position, and planned position for any MLC surpassing the ± 0.5 mm warning threshold. The warning thresholds were established based on the measured error ranges 95% confidence level: −0.12 to 0.38 mm for Y1 leaf and −0.24 to 0.27 mm for Y2 leaf, comparing log files against nominal values. Furthermore, the UI's right side displays the RMS and maximum MLC errors. A black horizontal line denotes the ± 0.5 mm warning threshold for visual reference. A clear PASS (green light) or WARNING (red light) indication is provided automatically after the analysis, signaling whether the machine has passed the virtual picket fence test. The results can be archived for longitudinal analysis after the evaluation.

**FIGURE 5:**
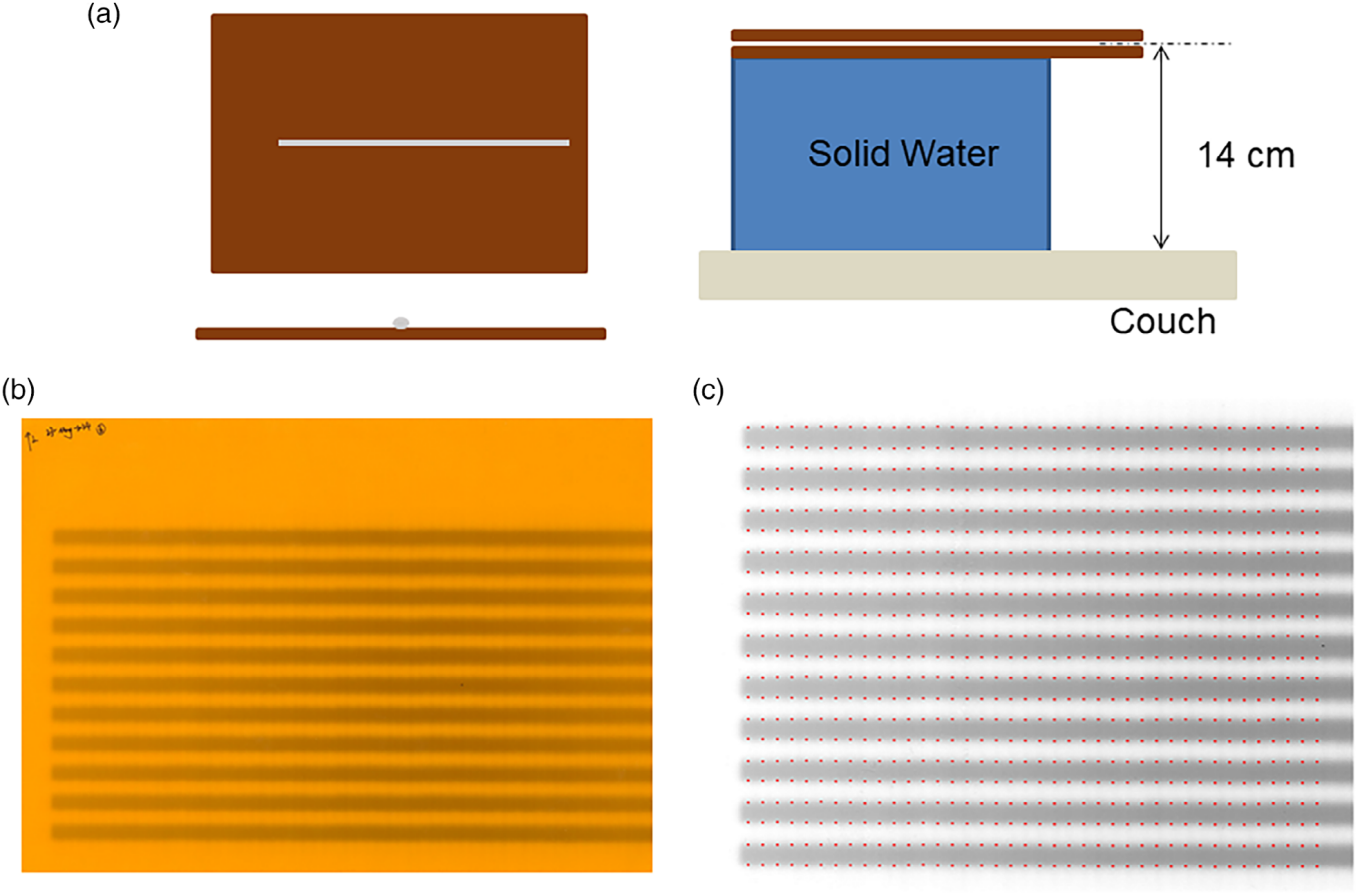
(a) The left side illustrates the isocenter definition prior to irradiation, with the gantry positioned in top‐down and lateral orientations, respectively. The dark rectangle represents the copper plate, while the silver line indicates the metal wire. The right side shows the setup for the picket fence test, where the film is elevated to the isocenter level using solid water pads. (b) The scanned film in RGB format. This film covers the left 40 pairs of MLCs. (c) The processed irradiated film using red channel data, where red dots represent the tips of various MLCs at different positions.

As illustrated in Figure 5b, the scanned film is in RGB format, but the analysis was based solely on the red channel. The pattern on the irradiated films appears blurrier compared to a conventional picket fence setup without copper plates, making it difficult to differentiate between different leaf pairs by eye. Therefore, we located each leaf based on its width and the distance from the isocenter marked on the film. In the longitudinal direction, the leaf position at each of the 11 segments was calculated using the longitudinal intensity curve. In Figure 5c, the detected leaf tip positions are marked as red dots on the processed film for the left 40 pairs of MLCs. Figure 7 presents the results of the comparison between leaf positions measured by film and the corresponding nominal values and log file data at T1. As shown in Figure 7a, the maximum deviation between the corresponding leaf positions on the film and their nominal values was within a 1 mm tolerance, with the RMS displayed in the right panel. Additionally, in Figure 7b, the maximum deviation between corresponding leaf positions on the film and in the log file was also within 1 mm for all leaves, with the RMS shown in the right panel. In the AQUA test, we analyzed the positioning accuracy for Y1 and Y2 leaves. The comparison between T1 and T5 using radiochromic film can be found in the supplementary materials.

**FIGURE 6:**
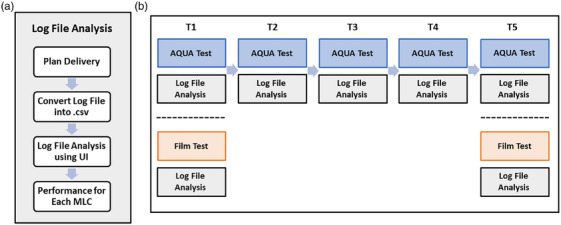
(a) Workflow for log file analysis in the virtual picket fence test. (b) The flowchart of the longitudinal analyses.

**FIGURE 7:**
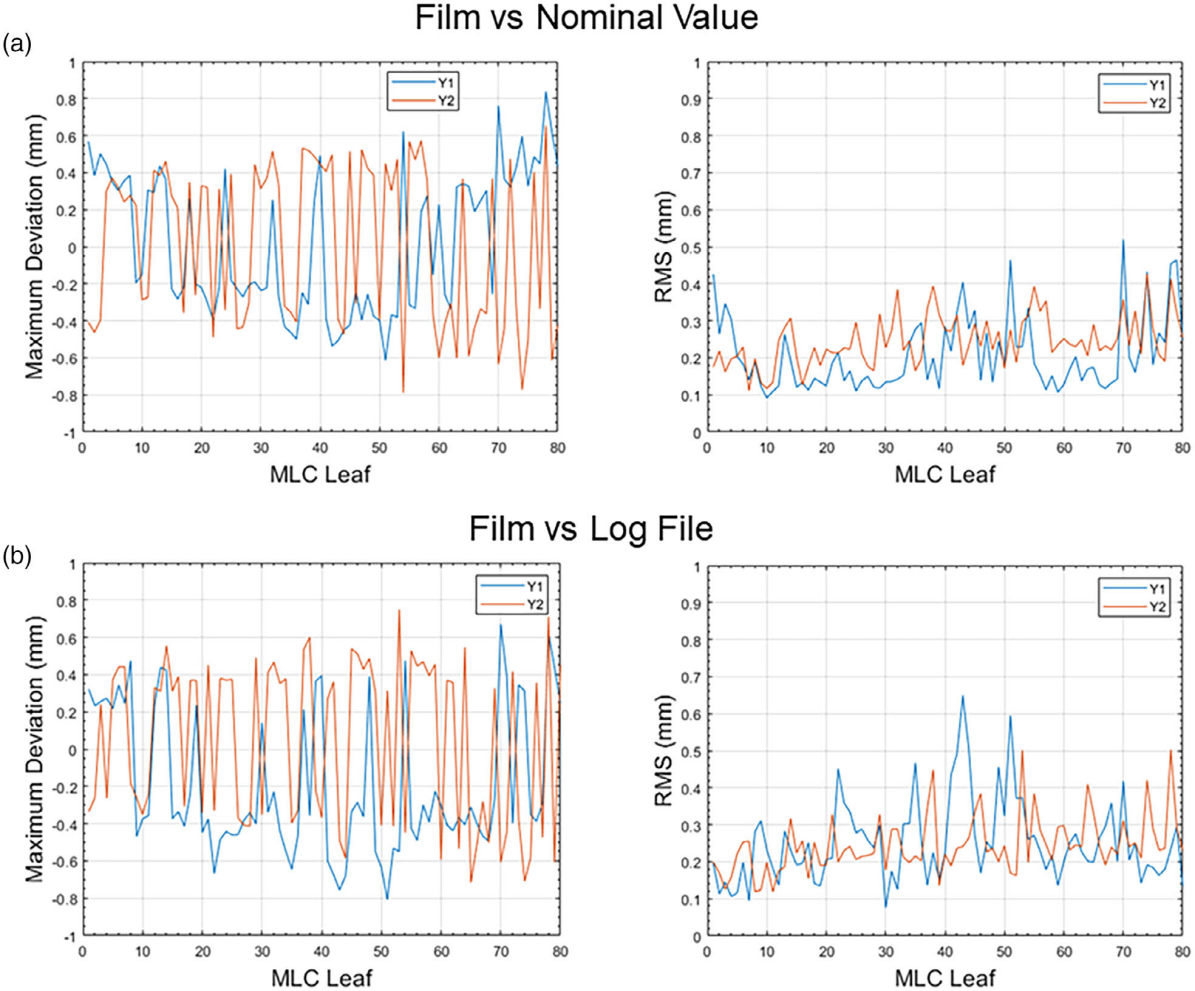
(a) The left panel shows the maximum leaf position deviation between the film and nominal values across 11 different positions for each leaf at T1. The right panel presents the RMS of the position deviation between the film and nominal values across the same 11 positions for each leaf. (b) The left panel shows the maximum leaf position deviation between the film and log file across 11 different positions for each leaf at T1. The right panel presents the RMS of the position deviation between the film and log file across these 11 positions for each leaf. RMS, root mean square.

**FIGURE 8:**
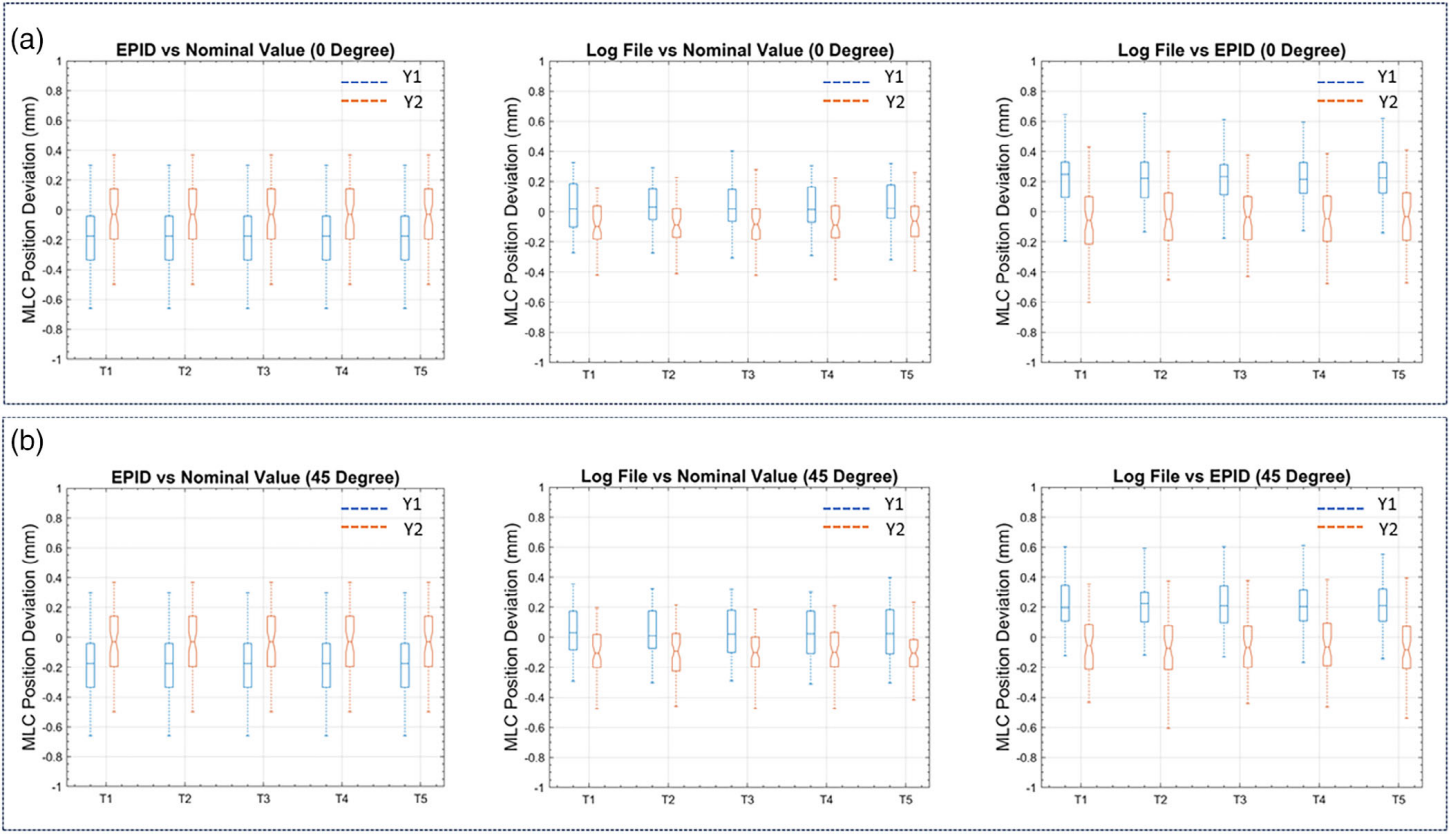
(a) Box plots illustrating the deviations among EPID, log file, and nominal values from T1 to T5 at 0° gantry angle. The central mark represents the median, while the bottom and top edges of the box indicate the 25th and 75th percentiles, respectively. Whiskers extend to the most extreme data points. (b) Box plots depicting the deviations among EPID, log file, and nominal values from T1 to T5 at 45° gantry angle.

Table 1 summarizes the leaf positions at T1 derived from EPID data and corresponding log files and nominal values at both 0° and 45°. For the Y1 leaf bank at 0°, the comparisons showed: EPID versus nominal positions (mean = −0.074 ± 0.22 mm; range: −0.46 to 0.31 mm), log file versus nominal positions (mean = 0.035 ± 0.16 mm; range: −0.27 to 0.33 mm), and log file versus EPID measurements (mean 0.11 ± 0.18 mm; range: −0.36 to 0.46 mm). For the Y2 leaf bank at 0°, the analysis showed: EPID versus nominal positions (mean = 0.14 ± 0.22 mm; range: −0.40 to 0.58 mm), log file versus nominal positions (mean = ‐0.084 ± 0.15 mm; range: −0.42 to 0.15 mm), and log file versus EPID measurements (mean = −0.23 ± 0.24 mm; range: −0.79 to 0.15 mm). For the Y1 leaf bank at 45°, the comparisons showed: EPID versus nominal positions (mean = −0.19 ± 0.20 mm; range: −0.66 to 0.30 mm), log file versus nominal positions (mean = 0.029 ± 0.17 mm; range: −0.29 to 0.32 mm), and log file versus EPID measurements (mean 0.22 ± 0.18 mm; range: −0.13 to 0.60 mm). For the Y2 leaf bank at 45°, the analysis showed: EPID versus nominal positions (mean = −0.34 ± 0.21 mm; range: −0.50 to 0.37 mm), log file versus nominal positions (mean = −0.10 ± 0.15 mm; range: −0.47 to 0.19 mm), and log file versus EPID measurements (mean = ‐0.070 ± 0.21 mm; range: −0.69 to 0.38 mm). Figure 8 presents a statistical analysis using box plots, demonstrating strong agreement between EPID, log file, and nominal values at both gantry orientations. This quantitative assessment revealed no significant drift or temporal fluctuations from T1 to T5. Mean positional errors remained consistently below 0.5 mm across all temporal data points.

**TABLE 1:** The comparison between EPID measurements and log files was performed for the central 28 leaf pairs at gantry angles of 0° and 45° at T1.

		EPID vs. Nominal Position	Log File vs. Nominal Position	Log File vs. EPID
0°	Y1	−0.074 ± 0.22 (‐0.46 ∼ 0.31)	0.035 ± 0.16 (−0.27 ∼ 0.33)	0.11 ± 0.18 (−0.36 to 0.46)
	Y2	0.14 ± 0.22 (−0.40 to 0.58)	−0.084 ± 0.15 (−0.42 to 0.15)	−0.23 ± 0.24 (−0.79 to 0.15)
45°	Y1	−0.19 ± 0.20 (−0.66 to 0.30)	0.029 ± 0.17 (−0.29 to 0.32)	0.22 ± 0.18 (−0.13 to 0.60)
	Y2	−0.34 ± 0.21 (−0.50 to 0.37)	−0.10 ± 0.15 (−0.47 to 0.19)	−0.070 ± 0.21(−0.69 to 0.38)

*Note*: The results are presented in terms of mean error, standard deviation, and error range, with all measurements reported in millimeters.

Abbreviation: EPID, electronic portal imaging devices.

## 4 DISCUSSION

This study presents a straightforward approach for conducting comprehensive MLC positioning checks, accompanied by a precise UI designed for physicists. We recommend integrating this test with the weekly AQUA procedure to evaluate MLC performance across all leaf pairs and throughout their full range of motion. This approach ensures thorough and efficient quality control of the MLC system.

Radiochromic film offers high spatial resolution and can accurately measure MLC positions.^15^ However, the largest available film size is insufficient to cover all MLCs of the Elekta Unity at the isocenter plane,^3^ which has a maximum field size of 57.4 cm × 22 cm. Consequently, two separate films are needed to cover all MLC pairs. Additionally, performing film‐based MLC checks in Unity is challenging due to the magnetic field's influence on secondary electron trajectories, potentially causing inaccuracies in MLC position determination on the film. To mitigate this, previous studies have used two copper plates to cover the film, reducing electron path length and ensuring consistent patterns on the irradiated film, unaffected by the magnetic field.^4^, ^6^, ^7^ Measuring the MLC accuracy at a gantry angle other than 0 degrees presents significant practical challenges. The complexity of positioning the film accurately at various gantry orientations makes such measurements nearly unfeasible in routine clinical settings. In conventional picket fence tests, inter‐leaf leakage creates a subtle contrast between adjacent leaves, helping define the edges of individual MLC leaves.^16^ However, the introduction of copper plates in the setup significantly alters this dynamic. The resulting irradiated film patterns become noticeably blurrier, complicating the visual identification of distinct leaf pairs. Leaf pairs in the transverse direction can only be distinguished based on their width and distance from the isocenter. Determination of the absolute distance from the leaf tip to the isocentric level requires localizing the isocenter position in the longitudinal direction as well. This process can be time‐consuming and may introduce errors when manually marking the isocenter on the film. Considering these limitations, conducting frequent film‐based MLC QA in Elekta Unity is challenging.

In our developed virtual picket fence test, neither field size nor magnetic field effects pose restrictions. The MLC positions are detected by the Rubicon optical leaf tracking technology.^17^ which is thoroughly calibrated during the commissioning process. Ultraviolet light illuminates ruby tips on the MLC leaves, generating infrared fluorescence. An infrared camera then detects this fluorescent signal to monitor and track leaf positions with real‐time precision. The scaled MLC positions at the isocenter plane are automatically stored in the log file. Unlike Linacs that rely on motor encoder counts for MLC positioning, the Elekta Unity MR‐Linac uses an optical leaf tracking system. This makes it less sensitive to mechanical issues like T‐nut wear or leaf backlash.^18^ By comparing the planned leaf positions with the actual leaf positions, we can evaluate the accuracy of all leaf pairs. This virtual picket fence test is easy to carry out, requiring no film or QA phantom. The use of log files for machine QA and patient‐specific QA is well established for conventional LINACs,^19^, ^20^ as well as for patient‐specific plan QA in Elekta Unity.^10^ However, no previous studies have utilized log files for routine MLC performance assessments in MR‐Linacs. In addition, this study examines the performance of individual leaves and offers a comprehensive longitudinal analysis of their trends. To further validate the accuracy of the detected positions in the log file, we conducted a comparison between the MLC positions recorded in the log file and those measured using radiochromic film at a 0‐degree gantry angle. The results demonstrated that the deviations for all MLCs between the film measurements and log file data were within a 1 mm tolerance. This close alignment between the log file data and measurement‐based QA provides strong evidence for the reliability of the log file in accurately representing MLC positions. Furthermore, the virtual picket fence test is more cost‐effective than film‐based methods. It eliminates phantom setup and can be completed in under 10 min, whereas film‐based tests require at least an hour for setup, exposure, and analysis. Additionally, the virtual test has no recurring material costs, such as film sheets. Besides, the virtual picket fence test shows comparable cost‐effectiveness with the AQUA test.

Several limitations should be noted. Log‐based MLC QA cannot detect transfer error. The leaf positions recorded in log files originate from the same system that controls leaf movement. The calibration process for Unity's MLC tracking system involves both leaf trajectory calibration and camera tilt and skew calibration, ensuring proper alignment of the imaging systems that monitor leaf positions. As a result, the accuracy of the optical tracking system is heavily reliant on the precision of these calibrations. This is an inevitable limitation of adopting log file for QA purposes. Any such calibration errors can be detected through monthly EPID or film‐based QA tests. In addition to the initial commissioning of the MLC leaf tracking system, comprehensive calibration of beam‐limiting devices, including the Rubicon optical system, is performed during preventive maintenance procedures conducted at 12‐month intervals. It is important to acknowledge that film measurements on the Elekta Unity introduce additional uncertainties compared to conventional Linacs, including potential inaccuracies during manual isocenter marking and reduced image resolution due to the copper plate. These factors increase the uncertainty in leaf position estimation using film, which may explain the observed discrepancies compared to EPID and log file data.

While the proposed virtual picket fence test offers valuable insights, it cannot entirely replace film‐based MLC checks, which provide direct visual confirmation of MLC positions. These complementary approaches work in tandem to ensure comprehensive QA of the MLC system. We recommend performing our proposed virtual picket fence test on a weekly basis,^21^ while maintaining less frequent film‐based MLC checks on a monthly basis. MLC performance at multiple gantry angles can be checked using our virtual picket fence test. This combined approach ensures comprehensive QA by leveraging the efficiency of the virtual test with the direct visualization provided by film‐based methods.

## 5 CONCLUSION

The proposed log file‐based virtual picket fence test offers a simple and practical solution for performing Elekta Unity MLC checks, enabling integration into the weekly QA workflow. Its implementation supports enhanced efficiency while maintaining rigorous MLC monitoring standards. We recommend its regular use in conjunction with traditional film‐based methods to ensure comprehensive quality control.

## AUTHOR CONTRIBUTIONS

Kai Yuan performed the experimental investigation, data analysis, and manuscript preparation. Matthew Manhin Cheung contributed to the experimental work and manuscript preparation. Louis Lee conceptualized the study and provided supervision. All authors reviewed and approved the final version.

## CONFLICT OF INTEREST STATEMENT

The authors declare no conflicts of interest.

## Supporting information



Supporting Information

## References

[1] Winkel D , Bol GH , Kroon PS , et al. Adaptive radiotherapy: the Elekta Unity MR‐linac concept. Clin Transl Radiat Oncol. 2019;18:54‐59. doi:10.1016/j.ctro.2019.04.001 31341976 PMC6630157

[2] Klein EE , Hanley J , Bayouth J , et al. Task Group 142 report: quality assurance of medical accelerators. Med Phys. 2009;36(9):4197‐4212. doi:10.1118/1.3190392 19810494

[3] Roberts DA , Sandin C , Vesanen PT , et al. Machine QA for the Elekta Unity system: a report from the Elekta MR‐linac consortium. Med Phys. 2021;48(5):e67‐e85. doi:10.1002/mp.14764 33577091 PMC8251771

[4] Snyder JE , St‐Aubin J , Yaddanapudi S , et al. Commissioning of a 1.5T Elekta Unity MR‐linac: a single institution experience. J Appl Clin Med Phys. 2020;21(7):160‐172. doi:10.1002/acm2.12902 32432405 PMC7386194

[5] Graves SA , Snyder JE , Boczkowski A , et al. Commissioning and performance evaluation of RadCalc for the Elekta unity MRI‐linac. J Appl Clin Med Phys. 2019;20(12):54‐62. doi:10.1002/acm2.12760 31722133 PMC6909114

[6] Tsuneda M , Abe K , Fujita Y , Ikeda Y , Furuyama Y , Uno T . Elekta Unity MR‐linac commissioning: mechanical and dosimetry tests. J Radiat Res (Tokyo). 2022;64(1):73‐84. doi:10.1093/jrr/rrac072

[7] van Zijp HM , van Asselen B , Wolthaus JWH , et al. Minimizing the magnetic field effect in MR‐linac specific QA‐tests: the use of electron dense materials. Phys Med Bio. 2016;61(3):N50. doi:10.1088/0031-9155/61/3/N50 26758570

[8] Sun S‐H , Kollitz E , Tseng W‐C , et al. MR‐linac MLC positioning QA by digitally stitching dual double‐exposed films. J Appl Clin Med Phys. 2024;25(7):e14325. doi:10.1002/acm2.14325 38467039 PMC11244661

[9] Chuang K‐C , Giles W , Adamson J . On the use of trajectory log files for machine & patient specific QA. Biomed Phys Eng Express. 2021;7(1):015010. doi:10.1088/2057-1976/abc86c 34037535

[10] Lim SB , Napolitano M , et al. An investigation of using log‐file analysis for automated patient‐specific quality assurance in MRgRT. J Appl Clin Med Phys. 2021;22(9):183‐188. doi:10.1002/acm2.13361 PMC8425925 34278711

[11] Sakaria K , Shaheera Midi N , Zin HM . Picket fence test of Agility MLCs using linac log data. J Phys Conf Ser. 2019;1248(1):012054. doi:10.1088/1742-6596/1248/1/012054

[12] Howard ME , Herman MG , Grams MP . Methodology for radiochromic film analysis using FilmQA Pro and ImageJ. PLoS One. 2020;15(5):e0233562. doi:10.1371/journal.pone.0233562 32437474 PMC7241712

[13] Méndez I , Rovira‐Escutia JJ , Casar B . A protocol for accurate radiochromic film dosimetry using Radiochromic.com. Radiol Oncol. 2021;55(3):369‐378. doi:10.2478/raon-2021-0034 34384012 PMC8366735

[14] Papaconstadopoulos P , Hegyi G , Seuntjens J , Devic S . A protocol for EBT3 radiochromic film dosimetry using reflection scanning. Med Phys. 2014;41(12):122101. doi:10.1118/1.4901308 25471974

[15] Rippke C , Renkamp CK , Attieh C , et al. Leaf‐individual calibration for a double stack multileaf collimator in photon radiotherapy. Phys Imaging Radiat Oncol. 2023;27:100477. doi:10.1016/j.phro.2023.100477 37635846 PMC10457557

[16] Li Y , Chen L , Zhu J , Wang B , Liu X . A quantitative method to the analysis of MLC leaf position and speed based on EPID and EBT3 film for dynamic IMRT treatment with different types of MLC. J Appl Clin Med Phys. 2017;18(4):106‐115. doi:10.1002/acm2.12102 PMC7663986 28517613

[17] Kabat CN , Defoor DL , Myers P , et al. Evaluation of the Elekta Agility MLC performance using high‐resolution log files. Med Phys. 2019;46(3):1397‐1407. doi:10.1002/mp.13374 30702748

[18] Barnes M , Pomare D , Doebrich M , et al. Insensitivity of machine log files to MLC leaf backlash and effect of MLC backlash on clinical dynamic MLC motion: an experimental investigation. J Appl Clin Med Phys. 2022;23(9):e13660. doi:10.1002/acm2.13660 35678793 PMC9512360

[19] Chow VUY , Kan MWK , Chan ATC . Patient‐specific quality assurance using machine log files analysis for stereotactic body radiation therapy (SBRT). J Appl Clin Med Phys. 2020;21(11):179‐187. doi:10.1002/acm2.13053 PMC7700944 33073897

[20] McGarry CK , Agnew CE , Hussein M , Tsang Y , Hounsell AR , Clark CH . The use of log file analysis within VMAT audits. Br J Radiol. 2016;89(1062). doi:10.1259/bjr.20150489 PMC5258140 27072390

[21] Kalavagunta C , Xu H , Zhang B , et al. Is a weekly qualitative picket fence test sufficient? A proposed alternate EPID‐based weekly MLC QA program. J Appl Clin Med Phys. 2022;23(8):e13699. doi:10.1002/acm2.13699 35856943 PMC9359020

