# Supplementary material for: Efficient MLC quality assurance using a virtual picket fence test in an MR‐Linac
Source: J Appl Clin Med Phys. 2025 Sep 3;26(9):e70232. doi: 10.1002/acm2.70232 (PMC12408376; doi:10.1002/acm2.70232)
Supplement: Supplementary file 1 — Supporting Information [file ACM2-26-e70232-s001.docx]

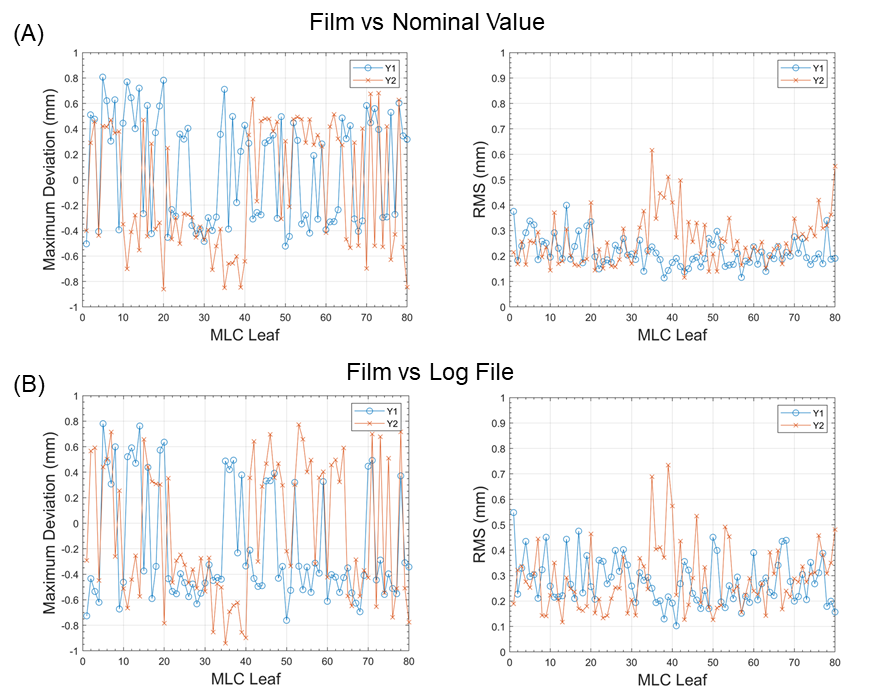


**Supplementary Figure 1.** (A) The left panel shows the maximum leaf position deviation between the film and nominal values across 11 different positions for each leaf at T5. The right panel presents the RMS of the position deviation between the film and nominal values across the same 11 positions for each leaf. (B) The left panel shows the maximum leaf position deviation between the film and log file across 11 different positions for each leaf at T5. The right panel presents the RMS of the position deviation between the film and log file across these 11 positions for each leaf.


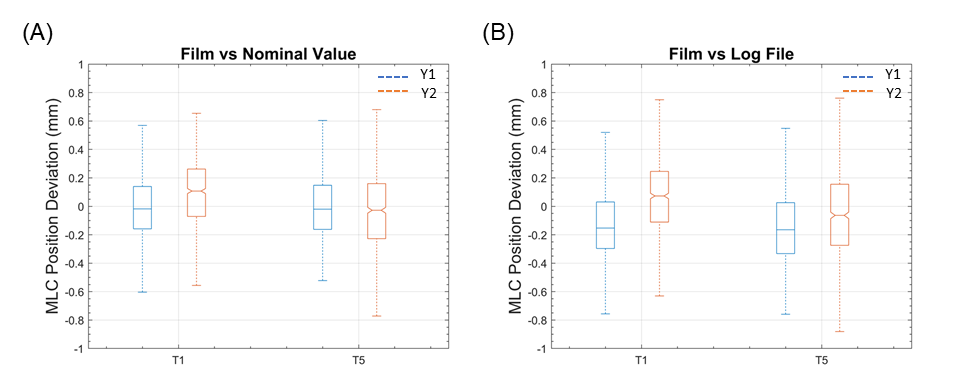


**Supplementary Figure 2.** (A) Statistical distribution of MLC deviations at T1 and T5 through box plot analysis, comparing film-derived data with nominal values. The central mark represents the median, while the bottom and top edges of the box indicate the 25th and 75th percentiles, respectively. Whiskers extend to the most extreme data points. (B) Statistical distribution of MLC deviations at T1 and T5 through box plot analysis, comparing film-derived data with log files.
